# The Skin Epilipidome in Stress, Aging, and Inflammation

**DOI:** 10.3389/fendo.2020.607076

**Published:** 2021-01-21

**Authors:** Florian Gruber, Martina Marchetti-Deschmann, Christopher Kremslehner, Markus Schosserer

**Affiliations:** ^1^ Christian Doppler Laboratory for Skin Multimodal Imaging of Aging and Senescence - SKINMAGINE -, Vienna, Austria; ^2^ Christian Doppler Laboratory on Biotechnology of Skin Aging, Vienna, Austria; ^3^ Department of Dermatology, Medical University of Vienna, Vienna, Austria; ^4^ Institute of Chemical Technologies and Analytics, TU Wien, Vienna, Austria; ^5^ Institute of Molecular Biotechnology, Department of Biotechnology, University of Natural Resources and Life Sciences, Vienna, Austria

**Keywords:** skin, ultraviolet, inflammation, stress, oxidized phospholipid, epilipidome, aging, senescence

## Abstract

Lipids are highly diverse biomolecules crucial for the formation and function of cellular membranes, for metabolism, and for cellular signaling. In the mammalian skin, lipids additionally serve for the formation of the epidermal barrier and as surface lipids, together regulating permeability, physical properties, acidification and the antimicrobial defense. Recent advances in accuracy and specificity of mass spectrometry have allowed studying enzymatic and non-enzymatic modifications of lipids—the epilipidome—multiplying the known diversity of molecules in this class. As the skin is an organ that is frequently exposed to oxidative-, chemical- and thermal stress, and to injury and inflammation, it is an ideal organ to study epilipidome dynamics, their causes, and their biological consequences. Recent studies uncover loss or gain in biological function resulting from either specific modifications or the sum of the modifications of lipids. These studies suggest an important role for the epilipidome in stress responses and immune regulation in the skin. In this minireview we provide a short survey of the recent developments on causes and consequences of epilipidomic changes in the skin or in cell types that reside in the skin.

## Introduction

The lipidome of keratinocytes (KC), the dominant cell type of the basal layer of the epidermis is made up mainly of phospholipids, cholesterol, and triacylglycerides. Differentiation of living KC into dead corneocytes, a controlled cell death process that continuously renews the epidermal barrier (1), drastically changes the KC’s lipid composition several times during the process. The last living (granular) epidermal layer contains cells with lamellar bodies containing glucosylceramides, phospholipids, and sphingomyelin which are further metabolized to produce the stratum corneum (SC) lipids, a mixture of free fatty acids (FFAs), cholesterol and ceramides (2, 3). The SC lipids form the lipid matrix, a flexible connection of low water permeability between the corneocytes which remain from terminal differentiation (4) and the FFAs contribute to the required acidification of the SC (5). Part of the surface lipids derive from the sebum, a mixture of TAG, wax esters, squalene and FFA, produced by holocrine secretion of terminally differentiating cells of the sebaceous gland, a lipid producing skin appendage. Most biological consequences of epilipidomic modification take place in the living layers of the epidermis or in the dermal compartment underneath; nonetheless SC lipids are susceptible to modifications. Some of these modifications are ROS-mediated (squalene oxidation), while others depend on enzymatic cascades, as for example in the formation of the lipid envelope where hydroxyl ceramides are esterified to corneocyte proteins by specific transglutaminases.

### Modification of The Skin Epilipidome by Ultraviolet Radiation

The best-studied oxidative modifier of skin lipids is solar radiation and wavelength bands thereof, which are used alone or in combination with photoactive chemicals as therapy for various skin diseases. The action of UV radiation (UVR) on human skin depends on wavelength and can induce acute inflammation-, immunosuppression, or cell death (6). The latter is elicited by combining UVR with photoactive drugs to specifically target cancer- or immune system cells. UVR can cause both enzymatic and non-enzymatic modification of lipids. The long-wavelength UVA (320–400 nm) oxidizes lipids in absence of enzymes (7, 8) but also shorter wavelength radiation can non-enzymatically generate oxidized lipids *via* free radical mechanisms (9). Cholesterol, phospholipids, free fatty acids, and squalene are targets for non-enzymatic lipid oxidation and yield bioactive products. Enzymatic synthesis of oxidized lipids, most prominently eicosanoids and related oxidized polyunsaturated fatty acids (PUFAs) results from UV activation of phospholipases, lipoxygenases and cyclooxygenases (10–12). Most of the work on enzymatic generation of eicosanoids [rev. in (10)] has been done on the response to clinically relevant short wavelength UVB irradiation. This may lead to an underestimation of non-enzymatic effects to solar UV exposure which are mostly elicited by longer wavelength radiation. Similarly biasing may be that UV-regulated eicosanoids (and related FA derived mediators) are investigated mainly in their free form, while a large fraction of the modified FA may be presently attached to more complex lipids.

Previously it was observed that the UVA-photo-oxidation of PUFA esterified to phospholipids is more efficient than photo-oxidation of the same PUFA in the free form, probably due to increased UVA induced singlet oxygen generation in the PL esterified configuration of the PUFA (13). Indeed, Leung et al. found in HaCaT cells exposed to UVA little effect on n-6 PUFA and their non-enzymatic oxidation products immediately after exposure (14) but detected elevation of enzymatically modified hydroxides of docosahexaenoic acid (DHA). The authors conclude that HaCaT cells required 24 h to return to PUFA homeostasis.

In primary human dermal fibroblasts, our group identified more than 500 features corresponding in retention properties to polar and oxidized phosphatidylcholines (PCs) that were induced immediately after irradiation with UVA (15), and also in primary human keratinocytes we found significant elevation of 173 OxPC species immediately after irradiation. In both cell types, the elevated species comprised also non-enzymatic PUFA-PC oxidation products such as PC-hydroperoxides and hydroxides, di-carboxylic and carbonyl group containing PC species. In the keratinocyte investigation we found that even at the high UVA-1 fluence of 40 J/cm² the cells recover, and most lipid species return to baseline levels within 24 h, insofar as the KC appear to limit especially the amount of highly reactive carbonyl containing lipids. The restoration of phospholipid redox (or epilipidome) homeostasis involves the antioxidant response, autophagy, the unfolded protein response and, as recent findings suggest, the transcriptional regulator NUPR1 (16). Conversely, in vitro oxidized PUFA-PC are potent inducers of autophagy and Nrf2 (17, 18). These are mechanisms and signaling pathways that can be assigned to the protective, pro-resolving spectrum of oxidized phospholipid action. At the same time these lipid extracts or *in-vitro* oxidized PAPC preparations contain phospholipids with known pro-inflammatory activity and highly reactive carbonyl compounds (19, 20). A detailed investigation of the quantities of individual lipid species and localization of the lipids, their functional groups and their adducts will be next steps for elucidating the biological net effect of epilipidomic modifications on (phospho-) lipids through oxidative stressors in the skin. Elaborate mass spectrometric methods are required for structural analysis of aldehyde adducts to proteins [rev in (21)]. Because even as antibodies to protein-lipid adducts and the dinitrophenylhydrazine method to investigate protein carbonylation are widely used, lipid oxidation products and especially malondialdehyde can show not only high diversity in the type of modification of proteins (and thereby yielding very different epitopes) (22), but also interfere with the detection of other adducts (23).

The dietary intake of fatty acids affects the systemic and cutaneous composition of systemic free fatty acids and the composition of phospholipids to which these fatty acids are dynamically esterified. It also affects the potential enzymatic and non-enzymatic oxidation products that will form after UV exposure. Supplementation with eicosapentaenoic acid (EPA) and a subsequent UV exposure led to a shift in the UVA induced eicosanoids that were recovered from skin suction blisters from arachidonic acid metabolites (prostaglandin E2 and 12-HETE) towards EPA metabolites (prostaglandin E3 and 12-hydroxy-eicosapentaenoic acid, respectively) which have less pro-inflammatory activity (24). When administering docosahexaenoic acid (DHA) to cultured fibroblasts, we observed an elevation of DHA-containing phospholipids which were highly susceptible to photo-oxidation. Only in Nrf2 deficient cells this increased oxidation susceptibility led to increased expression of inflammation markers. Therefore, both the type of UV-induced lipid signaling mediator and the cell’s capability to limit peroxidation may determine the epilipidomic effect on UV mediated inflammation regulation. UV not only can enzymatically generate immunomodulatory platelet activating factor (PAF), but PAF-like lipids can also result from free radical action on phospholipids. PAF and PAF-like lipids relay both acute inflammatory and delayed immunosuppressive UV effects, and potentially elicit systemic signals by releasing microvesicles from KC (25).

The effects of UV exposure are not restricted to cellular lipids. Also the sebum is susceptible to modification. Hydroperoxides of squalene generated by UV exposure have been identified *in vitro* and *in vivo* (26, 27), and as squalene is a major component of the epidermal surface lipids, its peroxidation products including also reactive aldehydes (28) were proposed as sensors conveying metabolic and inflammatory responses to UV radiation (29). One study even suggested that corneocyte dust containing high levels of oxidized squalene may be a relevant environmental irritant (30). The full spectrum of immunomodulatory actions of (UV-) oxidized squalene and other sebaceous lipids is discussed in (31), where the epidermal NLRP3 inflammasome is suggested as the cellular component that senses and relays inflammatory signaling.

An amplification of photo-damage is elicited by photosensitizers in photo(dynamic) therapy. Porphyrins and their derivatives have hydrophobic properties that locate them to membranes of target cells, allowing to kill those with light through photosensitized ROS generation. At the same time, this treatment leads to massive oxidation of (phospho) lipids (32), and it remains to be elucidated whether oxidized lipids interfere with- or contribute to the therapeutic efficacy. Lipotoxicity upon oxidative stress is mainly exerted by aldehydolipids and was reviewed in (33). In the skin context, the OxPL POVPC was toxic in melanocytes in the micromolar range (32), at which (34) we detected this lipid after exposure to physiologic fluences of UVA in other cell types (15).

## The Skin Epilipidome in Inflammation

The two major chronic inflammatory skin diseases associated with impaired barrier function, psoriasis and atopic dermatitis (AD), affect composition and ordering of the epidermal barrier lipids and composition of basal epidermal, dermal, and systemic lipids [reviewed in (10, 35, 36)]. Metabolites attributable to the epilipidome are regulated and likely contribute to the disease, but functional data are yet limited. 9- and 13-hydroxyoctadecadienoic acids (9- and 13-HODE) were significantly elevated in plasma samples from psoriatic patients, as was 7-hydroxycholesterol. In skin biopsies from the same patients the free and esterified levels of 8- and 12-hydroxy-eicosatetraenoic acids (8- and 12 HETE) and 9- and 13-HODE were accordingly elevated, but also eicosanoids with known anti-inflammatory properties (37). First data where resolvin D1 was applied on patient KC and reduced interleukin synthesis by these cells indicate that small pro-resolving mediators of the epilipidome that are topically applied or generated *in situ* could be useful for the treatment of psoriasis (38). At the same time the pro-inflammatory components of the epilipidome likely contribute to the inflammation. Interestingly, a phospholipase that is transferred *via* exosomes to Langerhans cells seems to process psoriasis specific antigens (39). Thus, clear spatial localization of lipid metabolites, *e.g.* with high resolution mass spectrometric imaging and detailed functional studies are needed to fully understand the contribution of the epilipidome in psoriasis.

In the sera of juvenile AD patients, leukotriene B4 (LTB4), thromboxane 2 (TXB2), prostaglandins, HETE and HODE were found elevated, and lipidomic analysis could distinguish between clinically relevant subgroups of patients with high *versus* low immunoglobulin E levels (40). Among the distinguishing markers lysophosphatidyl-ethanolamine (18:2), thromboxane b 2 (TXB2), and 11-, 12-dihydroxyeicosatrienoic acid (DHET) can be attributed to the epilipidome. TXB2 and 11, 12-DHET were found elevated in skin tissue lipid samples in a comparable study (41), that came to the conclusion that the ratio of pro-inflammatory to pro-resolution mediators was increased in the patients, especially PPARalpha agonistic oxidized lipids. These, especially 12-HETE mediate inflammation and disturb differentiation in AD organotypic skin models (42). Further research will elucidate the contribution of non-enzymatically formed isoforms or mimetics to the downstream signaling of these enzymatically generated mediators in skin inflammation. Agonism or signaling *via* prostaglandin receptors, PPARs, and pattern recognition receptors (PRR) through ROS mediated changes to lipids in other context has been reported (43–45).

## Modifications of The Skin Epilipidome by Exposure to Aging, Chemical Irritants, Drugs, and Other Stressors

Highly reactive lipid oxidation products and their adducts to other macromolecules accumulate in the skin that prematurely aged due to sun exposure (46, 47). However, also chronologic aging of the skin at the cellular level and senescence of cells are similarly associated with lipoxidizing redox events, for example ROS accumulation in mitochondrial dysfunction and in senescence related chronic inflammation (48). The skin’s cellular composition as well as the synthetic and metabolic fidelity changes during the mammalian lifespan, and these changes leave traces in the skin’s lipidome and epilipidome. Those epilipidomic changes introduce a novel, autonomous layer of signaling for complex exposure–response relationships (49) in cellular stress, aging, and inflammation. Recently, elevated leukotriene generation was identified as a feature of senescent fibroblasts that promotes lung fibrosis (50), and we found compatible changes in the oxidized phospholipidome of senescent dermal fibroblasts (51).

The skin is exposed to temperature fluctuations, which likely affects the dynamics of enzymatic- and ROS-mediated epilipidomic modifications. One study monitored barrier lipids of acne and control patients over the course of a year, together with trans-epidermal water loss (TEWL) measurements and assessment of acne severity. The authors found that in acne-affected skin the ceramide species Cer[NH] and Cer[AH] were significantly reduced. This effect was greatest in winter and correlated with the highest TEWL measurements. Ceramide species with 18-carbon species of 6-hydroxysphingosine appeared to be most significantly reduced, an example of the diverse consequences that oxidative modification of lipids has in epidermal barrier function (52). Compatible with the latter finding, a (redox-) lipidomic study (53) on SC lipids from volunteers receiving glucocorticosteroids (GC) identified that the barrier damage, which is a side effect of GC therapy, was associated with reduction of ceramides with an esterified omega-hydroxy acyl chain. Furthermore, anti-cancer chemotherapy can affect the skin epilipidome, shown in a murine melanoma model, where chemotherapy generated, probably due to ROS generation, PAF-receptor agonistic lipids which negatively affected anti-tumor immunity (54). In murine epidermis exposed to the carcinogenic chemical irritant 12-O-tetradecanoylphorbol 13-acetate (TPA), we found strong epilipidome modification. Phospholipid hydroperoxides were elevated three days after the last treatment, and we found that peroxiredoxin 6 is an important regulator of epidermal lipid (per) oxidation *in vivo* (55). Cigarette smoke (CS) is a lifestyle-related environmental stress for the skin, and exposure of KC to CS increases the formation of carbonyl (4-hydroxy-2-nonenal; 4-HNE) adducts which likely result in part from lipid oxidation (56), and the immunosuppressive PAF-like lipids (57). A novel therapeutic option for dermatological wound- and inflammation management is the directed application of beams of cold atmospheric plasma (CAP) which contains highly dynamic matter, to tissue (58). One consequence when this treatment is applied to surface lipids is a massive change in the skin epilipidome (59), and it remains to be investigated whether epilipidomic changes contribute to the efficacy of the treatment which appears to involve activation of the antioxidant response (60).

Whereas most of the studies discussed so far have investigated the modification of fatty acid residues, Maciel and colleagues reported that the radical generating 2,20-azobis(2-amidinopropane) dihydrochloride (AAPH) modifies the headgroup of phosphatidylserines in cultured keratinocytes, adding an additional layer of complexity and novel potential biological consequences to the epilipidome (61). Beyond the oxygen-mediated modifications to lipids, the complexity of the epilipidome can be increased by sulfonation of lipids (62) nitration and nitroxidation of phospholipids, observed *in vivo* in diabetes models and under metabolic stress [Rev. in (63)] and several nitro- and nitroso modifications of unsaturated PC and PS have been characterized (64). Nitro fatty acids were also found in dermal fibroblasts upon virus infection and impaired interferon gamma signaling (65) by modulating the palmitoylation of the adaptor molecule stimulator of IFN genes (STING) which led to inhibition of interferon release, and the authors suggested the pharmacological potential of these lipids in diseases caused by abnormally high STING activity.

## Discussion and Outlook—Connection of The Epilipidome With Other Non-Canonical Regulators and Localization of Epilipidomic Modifications Within The Skin

Although the importance of the epilipidome for the regulation of cellular processes is clearly evidenced (66), little is known about its interaction with other non-canonical regulators of cell fate (“epi-omics”), such as the epigenome, epitranscriptome, epiproteome or epimetabolome. As all of these “epi-omics” are influenced by oxidative stress, it is well conceivable that oxidized lipids further exacerbate the effects of the original redox stressor. For example, 4-HNE is formed by lipid peroxidation and is highly reactive towards cysteine, lysine and histidine residues. Thereby, protein adducts are formed which do not only impinge on the epiproteome (67), but also on the epigenome through covalent modification of histones. Histones are common advanced lipoxidation endproducts (ALEs), and some of them are associated with human disorders, such as systemic lupus erythematosus or Alzheimer’s disease (68). ALE formation impairs the interaction of histones with DNA and consequently leads to increased vulnerability of exposed DNA stretches to oxidative stress (69, 70). Similarly, chromatin reader, writer and eraser enzymes might be covalently modified by oxidized lipids and thereby their function might be altered. Besides histone acetylation, the epigenome is shaped by methyltransferases, adding methyl groups to bases of DNA. The metabolite S-adenosyl-methionine (SAM) might represent an important link between the different layers of “epi-omics”, because it acts as the universal methyl group donor for most DNA, RNA, lipid, and protein methylation reactions. Phospholipid methylation is the major consumer of SAM and SAM availability in cells is limited. Thus, changes in the methylation of phospholipids strongly reflect on methylation reactions of other substrates. Ye and colleagues provided evidence for this phenomenon by demonstrating that loss of phospholipid methylation causes hypermethylation of histones as well as of the major phosphatase PP2A (71). In contrast to DNA methylation, chemical modifications of different RNA species came into focus only recently (72), and might be subject to similar redox- and metabolism-based connections with the epilipidome (73–75). Moreover, RNA modifications were already implicated in the interaction of specific RNA molecules with lipid bilayers (76). N6-adenosine methylation of ribosomal RNA (rRNA) by METL-5 represents an interesting example for a complex crosstalk between the different layers of “epi-omics” in *Caenorhabditis elegans*. Methylation of A_1717_ on 18S rRNA enhances selective ribosomal binding and translation of CYP-29A3 mRNA. This enzyme is required for oxidation of eicosapentaenoic acid to eicosanoids and modulates heat stress resistance (77). Oxidized lipids might also directly influence selective protein synthesis through oxidation of ribosomal proteins (78). Since the synthesis of post-translational protein modifications, such as glycosylations, is tightly synchronized with translation, the epiproteome might be regulated by the epilipidome as well.

**Figure 1:**
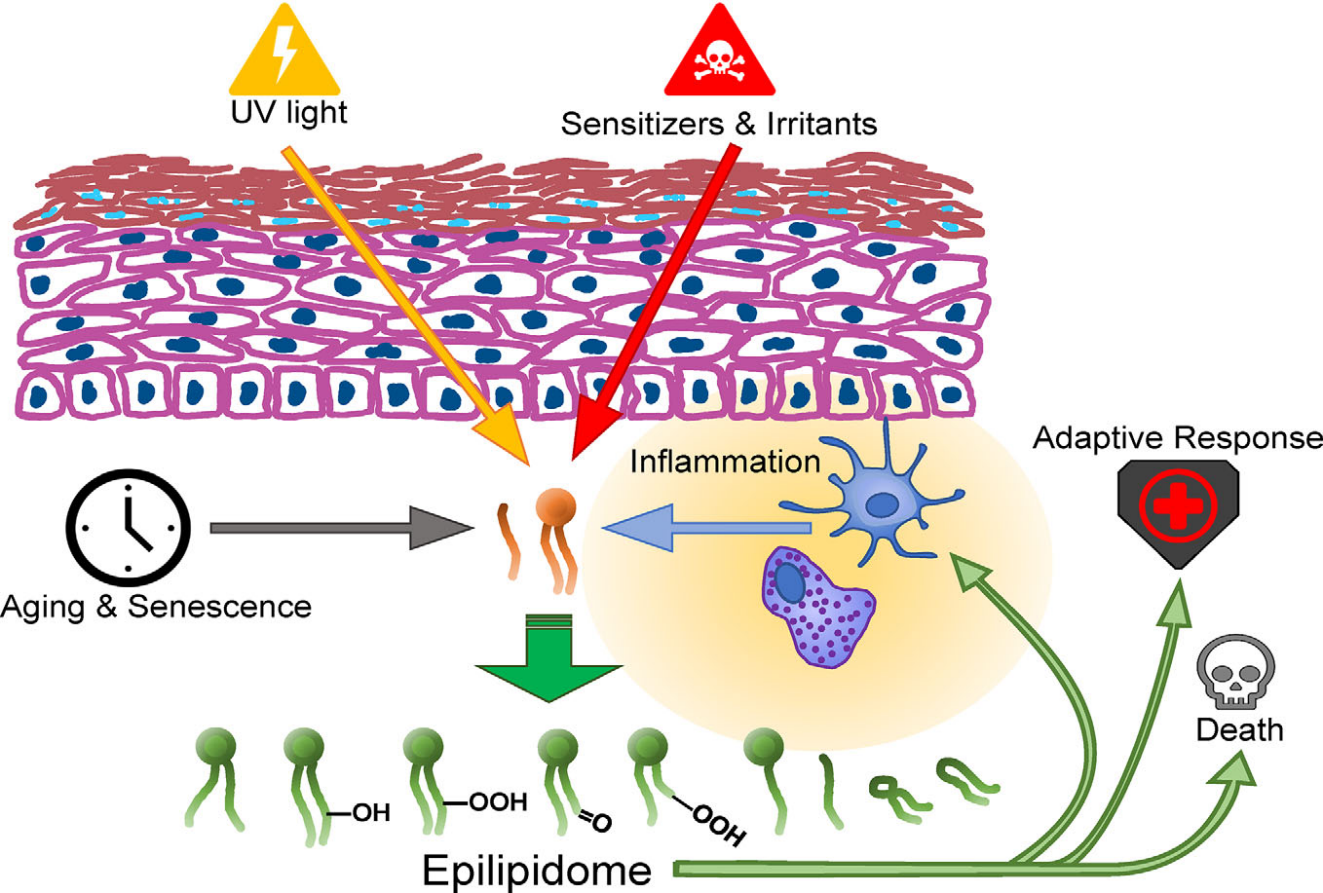
Formation routes and action spectrum of modified lipids in the skin.

The novel gold standard methods for redox- and other epilipidomic investigations are typically based on high resolution mass spectrometry (HRMS), often in combination with chromatographic separation and require intensive bioinformatic post-processing. These methods and their application on the lipidome, redoxlipidome and especially the skin are the topic of recent reviews that are suggested to the reader (35, 66, 79–85). The emerging technology of mass spectrometry-based imaging (MSI) has the unique feature to reveal the distribution of analytes within a tissue allowing the detection, localization and identification of multiple lipid species in an area of interest. Ionization techniques like secondary ion mass spectrometry (SIMS) (86), matrix assisted-laser desorption/ionization (MALDI) or desorption electrospray (DESI) (87) are the methods of choice allowing sensitive measurements. One tissue section can be used for consecutive measurements in positive and negative ion modes depending on the lipid class under investigation (88). However, low concentrations and ion suppression effects can lead to low ion intensities making lipid identification difficult. However, low signal intensities in respect to concentration levels of lipid peroxidation products or method-inherent ion suppression effects makes lipid identification by tandem MS often infeasible and HRMS (*i.e.* Fourier Transform Ion Cyclotron or Orbitrap) is indispensable. The novelty of MSI in the context of skin research is reflected by the limited number of publications available. Few papers focusing on sample preparation (89), few studies are available giving a general overview of lipid changes in skin during wound healing (90), in reconstructed skin equivalents (91) studying lipid profiles over time and in *ex vivo* human skin samples (92). Worth mentioning is research on the effect of topically applied compounds on lipid changes in the skin (93, 94). Despite the promising future of MS imaging, limitations have to be considered and challenges have to be met. One limitation is the rather low spatial resolution achieved with most instruments. (Nano)DESI provides spatial resolutions of approximately 40 to 100 µm, and conventional MALDI measurements can be carried out at pixel sizes down to 10 µm, still larger than most mammalian cells. As a result, each pixel represents the average lipid profile of maybe multiple cells and not of individual cells within the tissue. Reducing the spot size to a single cell level is therefore one of the most important endeavors in MSI research and instrument development (95). SIMS on the one hand has the potential to measure at a few nm spot size (approximately 30 nm), easily reaching cellular levels. However, SIMS is not a soft ionization technique, fragmenting lipid species and providing only lipid class information by head group analysis but not the full molecular information one is usually striving for. MALDI on the other hand is allowing the detection of intact lipid species at rather low resolution, being therefore the most often used method so far. But MALDI shows different ionization efficiencies for different lipid classes, making a comprehensive analysis for the entire lipidome a challenge, choosing the appropriate matrix is key (96). In summary, combining a multimodal approach at high spatial and mass resolution information on the skin’s epilipidome with immunohistological features of individual cells, their activation- and differentiation state, their metabolic configuration and their (epi-) transcriptome will be an important task in the imminent future that will help elucidate the contribution of the epilipidome to skin biology (**Figure 1**).

## Author Contributions

FG, MD, and MS wrote the manuscript. CK provided visualization. All authors contributed to the article and approved the submitted version.

## Funding

The financial support of the Federal Ministry for Digital and Economic Affairs (BMWFW) of Austria and the National Foundation for Research, Technology, and Development of Austria and of CHANEL Parfums et Beauté to the Christian Doppler Laboratory for Biotechnology of Skin Aging and the Christian Doppler Laboratory for Skin Multimodal Imaging of Aging and Senescence—SKINMAGINE—is gratefully acknowledged. The support of the Herzfelder’sche Familienstiftung, Austria, is gratefully acknowledged. These funding bodies do not issue grant numbers.

## Conflict of Interest

The authors declare that the research was conducted in the absence of any commercial or financial relationships that could be construed as a potential conflict of interest.

## References

[1] Eckhart L Zeeuwen PLJM The skin barrier: Epidermis vs environment. Exp Dermatol (2018) 27:805–6. 10.1111/exd.13731 29989217

[2] Feingold KR Elias PM Role of lipids in the formation and maintenance of the cutaneous permeability barrier. Biochim Biophys Acta (2014) 1841:280–94. 10.1016/j.bbalip.2013.11.007 24262790

[3] van Smeden J Janssens M Gooris GS Bouwstra JA The important role of stratum corneum lipids for the cutaneous barrier function. Biochim Biophys Acta (2014) 1841:295–313. 10.1016/j.bbalip.2013.11.006 24252189

[4] van Smeden J Bouwstra JA Stratum Corneum Lipids: Their Role for the Skin Barrier Function in Healthy Subjects and Atopic Dermatitis Patients. Curr Probl Dermatol (2016) 49:8–26. 10.1159/000441540 26844894

[5] Elias PM The how, why and clinical importance of stratum corneum acidification. Exp Dermatol (2017) 26:999–1003. 10.1111/exd.13329 28266738

[6] Bernard JJ Gallo RL Krutmann J Photoimmunology: how ultraviolet radiation affects the immune system. Nat Rev Immunol (2019) 19:688–701. 10.1038/s41577-019-0185-9 31213673

[7] Bose B Chatterjee SN UVA-induced peroxidation of lipid in the dried film state. J Photochem Photobiol B (1994) 23:119–23. 10.1016/1011-1344(94)06995-6 8040752

[8] Gruber F Oskolkova O Leitner A Mildner M Mlitz V Lengauer B Photooxidation generates biologically active phospholipids that induce heme oxygenase-1 in skin cells. J Biol Chem (2007) 282:16934–41. 10.1074/jbc.M702523200 17449870

[9] Konger RL Marathe GK Yao Y Zhang Q Travers JB Oxidized glycerophosphocholines as biologically active mediators for ultraviolet radiation-mediated effects. Prostaglandins Other Lipid Mediat (2008) 87(1–4):1–8. 10.1016/j.prostaglandins.2008.04.002 18555720 PMC3343742

[10] Kendall AC Nicolaou A Bioactive lipid mediators in skin inflammation and immunity. Prog Lipid Res (2013) 52:141–64. 10.1016/j.plipres.2012.10.003 23124022

[11] Niki E Lipid oxidation in the skin. Free Radic Res (2015) 49:827–34. 10.3109/10715762.2014.976213 25312699

[12] Wolfle U Seelinger G Bauer G Meinke MC Lademann J Schempp CM Reactive molecule species and antioxidative mechanisms in normal skin and skin aging. Skin Pharmacol Physiol (2014) 27:316–32. 10.1159/000360092 24994069

[13] Baier J Maisch T Regensburger J Pollmann C Baumler W Optical detection of singlet oxygen produced by fatty acids and phospholipids under ultraviolet A irradiation. J Biomed Opt (2008) 13:044029. 10.1117/1.2960553 19021356

[14] Leung KS Chan HF Leung HH Galano JM Oger C Durand T Short-time UVA exposure to human keratinocytes instigated polyunsaturated fatty acid without inducing lipid peroxidation. Free Radic Res (2017) 51:269–80. 10.1080/10715762.2017.1300885 28301979

[15] Gruber F Bicker W Oskolkova OV Tschachler E Bochkov VN A simplified procedure for semi-targeted lipidomic analysis of oxidized phosphatidylcholines induced by UVA irradiation. J Lipid Res (2012) 53:1232–42. 10.1194/jlr.D025270 PMC3351830 22414483

[16] Narzt MS Nagelreiter IM Oskolkova O Bochkov VN Latreille J Fedorova M A novel role for NUPR1 in the keratinocyte stress response to UV oxidized phospholipids. Redox Biol (2019) 20:467–82. 10.1016/j.redox.2018.11.006 PMC6243031 30466060

[17] Gruber F Mayer H Lengauer B Mlitz V Sanders JM Kadl A NF-E2-related factor 2 regulates the stress response to UVA-1-oxidized phospholipids in skin cells. FASEB J (2010) 24:39–48. 10.1096/fj.09-133520 19720622 PMC2797031

[18] Zhao Y Zhang CF Rossiter H Eckhart L Konig U Karner S Autophagy is induced by UVA and promotes removal of oxidized phospholipids and protein aggregates in epidermal keratinocytes. J Invest Dermatol (2013) 133:1629–37. 10.1038/jid.2013.26 23340736

[19] Bochkov V Gesslbauer B Mauerhofer C Philippova M Erne P Oskolkova OV Pleiotropic effects of oxidized phospholipids. Free Radic Biol Med (2017) 111:6–24. 10.1016/j.freeradbiomed.2016.12.034 28027924

[20] Bochkov VN Leitinger N Anti-inflammatory properties of lipid oxidation products. J Mol Med (2003) 81:613–26. 10.1007/s00109-003-0467-2 13679995

[21] Uchida K Aldehyde adducts generated during lipid peroxidation modification of proteins. Free Radic Res (2015) 49:896–904. 10.3109/10715762.2015.1036052 25968950

[22] Papac-Milicevic N Busch CJ Binder CJ Malondialdehyde Epitopes as Targets of Immunity and the Implications for Atherosclerosis. Adv Immunol (2016) 131:1–59. 10.1016/bs.ai.2016.02.001 27235680 PMC5892703

[23] Estevez M Padilla P Carvalho L Martin L Carrapiso A Delgado J Malondialdehyde interferes with the formation and detection of primary carbonyls in oxidized proteins. Redox Biol (2019) 26:101277. 10.1016/j.redox.2019.101277 31352127 PMC6669345

[24] Pilkington SM Rhodes LE Al-Aasswad NM Massey KA Nicolaou A Impact of EPA ingestion on COX- and LOX-mediated eicosanoid synthesis in skin with and without a pro-inflammatory UVR challenge–report of a randomised controlled study in humans. Mol Nutr Food Res (2014) 58:580–90. 10.1002/mnfr.201300405 PMC4377077 24311515

[25] Bihl JC Rapp CM Chen Y Travers JB UVB Generates Microvesicle Particle Release in Part Due to Platelet-activating Factor Signaling. Photochem Photobiol (2016) 92:503–6. 10.1111/php.12577 PMC5426906 26876152

[26] Ekanayake MS Hamburger M Elsner P Thiele JJ Ultraviolet a induces generation of squalene monohydroperoxide isomers in human sebum and skin surface lipids in vitro and in vivo. J Invest Dermatol (2003) 120:915–22. 10.1046/j.1523-1747.2003.12233.x 12787115

[27] Nakagawa K Ibusuki D Suzuki Y Yamashita S Higuchi O Oikawa S Ion-trap tandem mass spectrometric analysis of squalene monohydroperoxide isomers in sunlight-exposed human skin. J Lipid Res (2007) 48:2779–87. 10.1194/jlr.D700016-JLR200 17848584

[28] Dennis KJ Shibamoto T Production of malonaldehyde from squalene, a major skin surface lipid, during UV-irradiation. Photochem Photobiol (1989) 49:711–6. 10.1111/j.1751-1097.1989.tb08445.x 2756005

[29] Kostyuk V Potapovich A Stancato A De LC Lulli D Pastore S Photo-oxidation products of skin surface squalene mediate metabolic and inflammatory responses to solar UV in human keratinocytes. PLoS One (2012) 7:e44472. 10.1371/journal.pone.0044472 22952984 PMC3431355

[30] Fooshee DR Aiona PK Laskin A Laskin J Nizkorodov SA Baldi PF Atmospheric Oxidation of Squalene: Molecular Study Using COBRA Modeling and High-Resolution Mass Spectrometry. Environ Sci Technol (2015) 49:13304–13. 10.1021/acs.est.5b03552 26492333

[31] Oyewole AO Birch-Machin MA Sebum, inflammasomes and the skin: current concepts and future perspective. Exp Dermatol (2015) 24:651–4. 10.1111/exd.12774 26014614

[32] Melo T Santos N Lopes D Alves E Maciel E Faustino MA Photosensitized oxidation of phosphatidylethanolamines monitored by electrospray tandem mass spectrometry. J Mass Spectrom (2013) 48:1357–65. 10.1002/jms.3301 24338891

[33] Hauck AK Bernlohr DA Oxidative stress and lipotoxicity. J Lipid Res (2016) 57:1976–86. 10.1194/jlr.R066597 PMC5087875 27009116

[34] Ramprecht C Jaritz H Streith I Zenzmaier E Kofeler H Hofmann-Wellenhof R Toxicity of oxidized phosphatidylcholines in cultured human melanoma cells. Chem Phys Lipids (2015) 189:39–47. 10.1016/j.chemphyslip.2015.05.007 26028612

[35] Gruber F Kremslehner C Narzt MS The impact of recent advances in lipidomics and redox lipidomics on dermatological research. Free Radic Biol Med (2019) 144:256–65. 10.1016/j.freeradbiomed.2019.04.019 31004751

[36] Nicolaou A Eicosanoids in skin inflammation. Prostaglandins Leukot Essent Fatty Acids (2013) 88:131–8. 10.1016/j.plefa.2012.03.009 22521864

[37] Sorokin AV Domenichiello AF Dey AK Yuan ZX Goyal A Rose SM Bioactive Lipid Mediator Profiles in Human Psoriasis Skin and Blood. J Invest Dermatol (2018) 138:1518–28. 10.1016/j.jid.2018.02.003 PMC6121727 29454560

[38] Sorokin AV Norris PC English JT Dey AK Chaturvedi A Baumer Y Identification of proresolving and inflammatory lipid mediators in human psoriasis. J Clin Lipidol (2018) 12:1047–60. 10.1016/j.jacl.2018.03.091 PMC6112609 29730187

[39] Cheung KL Jarrett R Subramaniam S Salimi M Gutowska-Owsiak D Chen YL Psoriatic T cells recognize neolipid antigens generated by mast cell phospholipase delivered by exosomes and presented by CD1a. J Exp Med (2016) 213:2399–412. 10.1084/jem.20160258 PMC5068234 27670592

[40] Huang Y Chen G Liu X Shao Y Gao P Xin C Serum metabolomics study and eicosanoid analysis of childhood atopic dermatitis based on liquid chromatography-mass spectrometry. J Proteome Res (2014) 13:5715–23. 10.1021/pr5007069 25316199

[41] Torocsik D Weise C Gericke J Szegedi A Lucas R Mihaly J Transcriptomic and lipidomic profiling of eicosanoid / docosanoid signalling in affected and non-affected skin of human atopic dermatitis patients. Exp Dermatol (2018) 28(2):177–89. 10.1111/exd.13867 30575130

[42] Blunder S Ruhl R Moosbrugger-Martinz V Krimmel C Geisler A Zhu H Alterations in Epidermal Eicosanoid Metabolism Contribute to Inflammation and Impaired Late Differentiation in FLG-Mutated Atopic Dermatitis. J Invest Dermatol (2017) 137:706–15. 10.1016/j.jid.2016.09.034 PMC5551680 27793761

[43] Delerive P Furman C Teissier E Fruchart J Duriez P Staels B Oxidized phospholipids activate PPARalpha in a phospholipase A2-dependent manner. FEBS Lett (2000) 471:34–8. 10.1016/S0014-5793(00)01364-8 10760508

[44] Itoh T Fairall L Amin K Inaba Y Szanto A Balint BL Structural basis for the activation of PPARgamma by oxidized fatty acids. Nat Struct Mol Biol (2008) 15:924–31. 10.1038/nsmb.1474 PMC2939985 19172745

[45] Li R Mouillesseaux KP Montoya D Cruz D Gharavi N Dun M Identification of prostaglandin E2 receptor subtype 2 as a receptor activated by OxPAPC. Circ Res (2006) 98:642–50. 10.1161/01.RES.0000207394.39249.fc 16456101

[46] Larroque-Cardoso P Camare C Nadal-Wollbold F Grazide MH Pucelle M Garoby-Salom S Elastin Modification by 4-Hydroxynonenal in Hairless Mice Exposed to UV-A. Role in Photoaging and Actinic Elastosis. J Invest Dermatol (2015) 135:1873–81. 10.1038/jid.2015.84 25739050

[47] Williams JD Bermudez Y Park SL Stratton SP Uchida K Hurst CA Malondialdehyde-derived epitopes in human skin result from acute exposure to solar UV and occur in nonmelanoma skin cancer tissue. J Photochem Photobiol B (2014) 132:56–65. 10.1016/j.jphotobiol.2014.01.019 24584085 PMC3973651

[48] Gruber F Kremslehner C Eckhart L Tschachler E Cell aging and cellular senescence in skin aging - Recent advances in fibroblast and keratinocyte biology. Exp Gerontol (2020) 130:110780. 10.1016/j.exger.2019.110780 31794850

[49] Ghezzi P Floridi L Boraschi D Cuadrado A Manda G Levic S Oxidative Stress and Inflammation Induced by Environmental and Psychological Stressors: A Biomarker Perspective. Antioxid Redox Signal (2018) 28:852–72. 10.1089/ars.2017.7147 28494612

[50] Wiley CD Brumwell AN Davis SS Jackson JR Valdovinos A Calhoun C Secretion of leukotrienes by senescent lung fibroblasts promotes pulmonary fibrosis. JCI Insight (2019) 4(24):e130056. 10.1172/jci.insight.130056 PMC6975274 31687975

[51] Narzt MS Pils V Kremslehner C Nagelreiter IM Schosserer M Bessonova E Epilipidomics of senescent dermal fibroblasts identify lysophosphatidylcholines as pleiotropic SASP factors. J Invest Dermatol (2020) S0022-202X(20)32367-8. 10.1016/j.jid.2020.11.020 33333126

[52] Pappas A Kendall AC Brownbridge LC Batchvarova N Nicolaou A Seasonal changes in epidermal ceramides are linked to impaired barrier function in acne patients. Exp Dermatol (2018) 27:833–6. 10.1111/exd.13499 29356138

[53] Ropke MA Alonso C Jung S Norsgaard H Richter C Darvin ME Effects of glucocorticoids on stratum corneum lipids and function in human skin-A detailed lipidomic analysis. J Dermatol Sci (2017) 88:330–8. 10.1016/j.jdermsci.2017.08.009 28911799

[54] Sahu RP Ocana JA Harrison KA Ferracini M Touloukian CE Al-Hassani M Chemotherapeutic agents subvert tumor immunity by generating agonists of platelet-activating factor. Cancer Res (2014) 74:7069–78. 10.1158/0008-5472.CAN-14-2043 PMC4252249 25304264

[55] Rolfs F Huber M Gruber F Bohm F Pfister HJ Bochkov VN Dual role of the antioxidant enzyme peroxiredoxin 6 in skin carcinogenesis. Cancer Res (2013) 73:3460–9. 10.1158/0008-5472.CAN-12-4369 23576553

[56] Sticozzi C Belmonte G Pecorelli A Arezzini B Gardi C Maioli E Cigarette smoke affects keratinocytes SRB1 expression and localization via H2O2 production and HNE protein adducts formation. PLoS One (2012) 7:e33592. 10.1371/journal.pone.0033592 22442701 PMC3307738

[57] Sahu RP Petrache I Van Demark MJ Rashid BM Ocana JA Tang Y Cigarette smoke exposure inhibits contact hypersensitivity via the generation of platelet-activating factor agonists. J Immunol (2013) 190:2447–54. 10.4049/jimmunol.1202699 PMC3577966 23355733

[58] Heinlin J Isbary G Stolz W Morfill G Landthaler M Shimizu T Plasma applications in medicine with a special focus on dermatology. J Eur Acad Dermatol Venereol (2011) 25:1–11. 10.1111/j.1468-3083.2010.03702.x 20497290

[59] Striesow J Lackmann JW Ni Z Wenske S Weltmann KD Fedorova M Oxidative modification of skin lipids by cold atmospheric plasma (CAP): A standardizable approach using RP-LC/MS(2) and DI-ESI/MS(2). Chem Phys Lipids (2020) 226:104786. 10.1016/j.chemphyslip.2019.104786 31229410

[60] Schmidt A Bekeschus S Redox for Repair: Cold Physical Plasmas and Nrf2 Signaling Promoting Wound Healing. Antioxidants (Basel) (2018) 7(10):146. 10.3390/antiox7100146 PMC6210784 30347767

[61] Maciel E Neves BM Santinha D Reis A Domingues P Teresa CM Detection of phosphatidylserine with a modified polar head group in human keratinocytes exposed to the radical generator AAPH. Arch Biochem Biophys (2014) 548:38–45. 10.1016/j.abb.2014.02.002 24560783

[62] Dias IH Ferreira R Gruber F Vitorino R Rivas-Urbina A Sanchez-Quesada JL Sulfate-based lipids: analysis of healthy human fluids and cell extracts. Chem Phys Lipids (2019) 221:53–64. 10.1016/j.chemphyslip.2019.03.009 30910732

[63] Melo T Domingues P Ferreira R Milic I Fedorova M Santos SM Recent Advances on Mass Spectrometry Analysis of Nitrated Phospholipids. Anal Chem (2016) 88(5):2622–9. 10.1021/acs.analchem.5b03407 26814598

[64] Neves B Domingues P Oliveira MM Domingues MDR Melo T Profile of Phosphatidylserine Modifications under Nitroxidative Stress Conditions Using a Liquid Chromatography-Mass Spectrometry Based Approach. Molecules (2018) 24(1):107. 10.3390/molecules24010107 PMC6337642 30597957

[65] Hansen AL Buchan GJ Ruhl M Mukai K Salvatore SR Ogawa E Nitro-fatty acids are formed in response to virus infection and are potent inhibitors of STING palmitoylation and signaling. Proc Natl Acad Sci USA (2018) 115:E7768–75. 10.1073/pnas.1806239115 PMC6099880 30061387

[66] Ni Z Goracci L Cruciani G Fedorova M Computational solutions in redox lipidomics - Current strategies and future perspectives. Free Radic Biol Med (2019) 144:110–23. 10.1016/j.freeradbiomed.2019.04.027 31035005

[67] Griesser E Vemula V Raulien N Wagner U Reeg S Grune T Cross-talk between lipid and protein carbonylation in a dynamic cardiomyocyte model of mild nitroxidative stress. Redox Biol (2017) 11:438–55. 10.1016/j.redox.2016.12.028 PMC5226815 28086193

[68] Kreuz S Fischle W Oxidative stress signaling to chromatin in health and disease. Epigenomics (2016) 8:843–62. 10.2217/epi-2016-0002 PMC5619053 27319358

[69] Drake J Petroze R Castegna A Ding Q Keller JN Markesbery WR 4-Hydroxynonenal oxidatively modifies histones: implications for Alzheimer’s disease. Neurosci Lett (2004) 356:155–8. 10.1016/j.neulet.2003.11.047 15036618

[70] Garcia-Gimenez JL Roma-Mateo C Pallardo FV Oxidative post-translational modifications in histones. Biofactors (2019) 45:641–50. 10.1002/biof.1532 31185139

[71] Ye C Sutter BM Wang Y Kuang Z Tu BP A Metabolic Function for Phospholipid and Histone Methylation. Mol Cell (2017) 66:180–93. 10.1016/j.molcel.2017.02.026 PMC5482412 28366644

[72] Song J Yi C Chemical Modifications to RNA: A New Layer of Gene Expression Regulation. ACS Chem Biol (2017) 12:316–25. 10.1021/acschembio.6b00960 28051309

[73] Li Z Chen X Liu Z Ye W Li L Qian L Recent Advances: Molecular Mechanism of RNA Oxidation and Its Role in Various Diseases. Front Mol Biosci (2020) 7:184. 10.3389/fmolb.2020.00184 32850971 PMC7413073

[74] Nunomura A Lee HG Zhu X Perry G Consequences of RNA oxidation on protein synthesis rate and fidelity: implications for the pathophysiology of neuropsychiatric disorders. Biochem Soc Trans (2017) 45:1053–66. 10.1042/BST20160433 28778984

[75] Thomas JM Batista PJ Meier JL Metabolic Regulation of the Epitranscriptome. ACS Chem Biol (2019) 14:316–24. 10.1021/acschembio.8b00951 30653309

[76] Janas T Janas T Yarus M Human tRNA(Sec) associates with HeLa membranes, cell lipid liposomes, and synthetic lipid bilayers. RNA (2012) 18:2260–8. 10.1261/rna.035352.112 PMC3504676 23097422

[77] Liberman N O’Brown ZK Earl AS Boulias K Gerashchenko MV Wang SY N6-adenosine methylation of ribosomal RNA affects lipid oxidation and stress resistance. Sci Adv (2020) 6:eaaz4370. 10.1126/sciadv.aaz4370 32494643 PMC7176415

[78] Shcherbik N Pestov DG The Impact of Oxidative Stress on Ribosomes: From Injury to Regulation. Cells (2019) 8(11):1379. 10.3390/cells8111379 PMC6912279 31684095

[79] Astarita G Kendall AC Dennis EA Nicolaou A Targeted lipidomic strategies for oxygenated metabolites of polyunsaturated fatty acids. Biochim Biophys Acta (2015) 1851:456–68. 10.1016/j.bbalip.2014.11.012 PMC4323855 25486530

[80] Brugger B Lipidomics: analysis of the lipid composition of cells and subcellular organelles by electrospray ionization mass spectrometry. Annu Rev Biochem (2014) 83:79–98. 10.1146/annurev-biochem-060713-035324 24606142

[81] Hu C Wang M Han X Shotgun lipidomics in substantiating lipid peroxidation in redox biology: Methods and applications. Redox Biol (2017) 12:946–55. 10.1016/j.redox.2017.04.030 PMC5423350 28494428

[82] Kendall AC Koszyczarek MM Jones EA Hart PJ Towers M Griffiths CEM Lipidomics for translational skin research: A primer for the uninitiated. Exp Dermatol (2018) 27:721–8. 10.1111/exd.13558 29654617

[83] Li S Ganguli-Indra G Indra AK Lipidomic analysis of epidermal lipids: a tool to predict progression of inflammatory skin disease in humans. Expert Rev Proteomics (2016) 13:451–6. 10.1080/14789450.2016.1177462 PMC4939172 27121756

[84] Massey KA Nicolaou A Lipidomics of oxidized polyunsaturated fatty acids. Free Radic Biol Med (2013) 59:45–55. 10.1016/j.freeradbiomed.2012.08.565 22940496 PMC3991857

[85] Spickett CM Pitt AR Oxidative lipidomics coming of age: advances in analysis of oxidized phospholipids in physiology and pathology. Antioxid Redox Signal (2015) 22:1646–66. 10.1089/ars.2014.6098 PMC4486145 25694038

[86] Passarelli MK Winograd N Lipid imaging with time-of-flight secondary ion mass spectrometry (ToF-SIMS). Biochim Biophys Acta (2011) 1811:976–90. 10.1016/j.bbalip.2011.05.007 PMC3199347 21664291

[87] Gode D Volmer DA Lipid imaging by mass spectrometry - a review. Analyst (2013) 138:1289–315. 10.1039/c2an36337b 23314100

[88] Berry KA Hankin JA Barkley RM Spraggins JM Caprioli RM Murphy RC MALDI imaging of lipid biochemistry in tissues by mass spectrometry. Chem Rev (2011) 111:6491–512. 10.1021/cr200280p PMC3199966 21942646

[89] de Macedo CS Anderson DM Schey KL MALDI (matrix assisted laser desorption ionization) Imaging Mass Spectrometry (IMS) of skin: Aspects of sample preparation. Talanta (2017) 174:325–35. 10.1016/j.talanta.2017.06.018 28738588

[90] Castellanos A Hernandez MG Tomic-Canic M Jozic I Fernandez-Lima F Multimodal, in Situ Imaging of Ex Vivo Human Skin Reveals Decrease of Cholesterol Sulfate in the Neoepithelium during Acute Wound Healing. Anal Chem (2020) 92:1386–94. 10.1021/acs.analchem.9b04542 PMC8341291 31789498

[91] Mitchell CA Long H Donaldson M Francese S Clench MR Lipid changes within the epidermis of living skin equivalents observed across a time-course by MALDI-MS imaging and profiling. Lipids Health Dis (2015) 14:84. 10.1186/s12944-015-0089-z 26243140 PMC4525729

[92] Hart PJ Clench MR MALDI-MSI of Lipids in Human Skin. Methods Mol Biol (2017) 1618:29–36. 10.1007/978-1-4939-7051-3_4 28523497

[93] Mitchell CA Donaldson M Francese S Clench MR MALDI MSI analysis of lipid changes in living skin equivalents in response to emollient creams containing palmitoylethanolamide. Methods (2016) 104:93–100. 10.1016/j.ymeth.2016.02.001 26845462

[94] Sjovall P Skedung L Gregoire S Biganska O Clement F Luengo GS Imaging the distribution of skin lipids and topically applied compounds in human skin using mass spectrometry. Sci Rep (2018) 8:16683. 10.1038/s41598-018-34286-x 30420715 PMC6232133

[95] Bowman AP Bogie JFJ Hendriks JJA Haidar M Belov M Heeren RMA Evaluation of lipid coverage and high spatial resolution MALDI-imaging capabilities of oversampling combined with laser post-ionisation. Anal Bioanal Chem (2020) 412:2277–89. 10.1007/s00216-019-02290-3 PMC7118047 31879798

[96] Leopold J Popkova Y Engel KM Schiller J Recent Developments of Useful MALDI Matrices for the Mass Spectrometric Characterization of Lipids. Biomolecules (2018) 8(4):173 10.3390/biom8040173 PMC6316665 30551655

